# Broadband Energy Harvester Using Non-linear Polymer Spring and Electromagnetic/Triboelectric Hybrid Mechanism

**DOI:** 10.1038/srep41396

**Published:** 2017-01-25

**Authors:** Rahul Kumar Gupta, Qiongfeng Shi, Lokesh Dhakar, Tao Wang, Chun Huat Heng, Chengkuo Lee

**Affiliations:** 1 Department of Electrical and Computer Engineering, National University of Singapore, 4 Engineering Drive 3, 117576, Singapore; 2 Center for Intelligent Sensors and MEMS (CISM), National University of Singapore, Singapore; 3 NUS Suzhou Research Institute (NUSRI), Suzhou Industrial Park, Suzhou 215123, P. R. China; 4 NUS Graduate School for Integrative Science and Engineering, National University of Singapore, 117456, Singapore

## Abstract

Over the years, several approaches have been devised to widen the operating bandwidth, but most of them can only be triggered at high accelerations. In this work, we investigate a broadband energy harvester based on combination of non-linear stiffening effect and multimodal energy harvesting to obtain high bandwidth over wide range of accelerations (0.1 g–2.0 g). In order to achieve broadband behavior, a polymer based spring exhibiting multimodal energy harvesting is used. Besides, non-linear stiffening effect is introduced by using mechanical stoppers. At low accelerations (<0.5 g), the nearby mode frequencies of polymer spring contribute to broadening characteristics, while proof mass engages with mechanical stoppers to introduce broadening by non-linear stiffening at higher accelerations. The electromagnetic mechanism is employed in this design to enhance its output at low accelerations when triboelectric output is negligible. Our device displays bandwidth of 40 Hz even at low acceleration of 0.1 g and it is increased up to 68 Hz at 2 g. When non-linear stiffening is used along with multimodal energy-harvesting, the obtained bandwidth increases from 23 Hz to 68 Hz with percentage increment of 295% at 1.8 g. Further, we have demonstrated the triboelectric output measured as acceleration sensing signals in terms of voltage and current sensitivity of 4.7 Vg^−1^ and 19.7 nAg^−1^, respectively.

Over the past few years, energy harvesters from body heat^1^,^2^,^3^,^4^,^5^ and ambient available vibrations^6^,^7^,^8^,^9^,^10^,^11^,^12^ such as human-walking, machine-vibrations, robotic movements, wind, ocean waves and aircrafts, etc. have been widely investigated as means to replace the battery in a system or to realize self-powered sensors^13^,^14^,^15^,^16^,^17^,^18^,^19^,^20^,^21^. To realize the self-powered sensors, piezoelectric energy harvesting mechanisms have been comprehensively explored because of the direct piezoelectric effect, i.e., charge output caused by strain or deformation on PZT, ZnO and AlN ceramics and poly(vinylidene fluoride) (PVDF) polymer films^22^,^23^,^24^,^25^. A piezoelectric PZT microcantilever has been studied as air flow sensors and wind-driven energy harvesters with flow sensing sensitivity of 0.9 mV/(m/s) and electrical output power of 3.3 nW at load resistance of 100 KΩ and flow velocity of 15.6 m/s, respectively^26^,^27^. Implantable PZT thin film, ZnO nanowires and PVDF patches have been reported as self-powered strain (or pulse) sensors to harvest vibration energy from heart and lung^7^,^28^,^29^,^30^. Recently piezoelectric energy harvesters have been integrated with neural electrodes for brain and nerves stimulation^31^,^32^.

Parallel to progress in the self-powered sensor research, various approaches have been explored to scavenge mechanical kinetic energy associated with human-walking, body stretching, head/hand-shaking and finger moving or bending, etc^33^,^34^,^35^,^36^,^37^,^38^. Among these approaches, vibration energy harvesters (VEHs) receive majority of research interests and have been demonstrated by using one of piezoelectric^39^,^40^,^41^,^42^, electrostatic^43^,^44^,^45^,^46^, electromagnetic^47^,^48^,^49^,^50^,^51^,^52^ and triboelectric^53^,^54^,^55^,^56^,^57^ mechanisms. In addition to self-powered piezoelectric sensors, there are some self-powered triboelectric sensors reported to detect force, pressure, tactile, displacement, vibration, liquid volume, liquid flow, ion concentration, and organic concentration, etc^58^,^59^,^60^,^61^,^62^,^63^,^64^,^65^,^66^,^67^,^68^,^69^. General speaking, when two triboelectrically opposite materials come in contact, they generate equal and opposite charges according to their place in the triboelectric series. The flexible triboelectric nanogenerator (TENG) consists of two polymer films, which have different electron-attracting abilities, with metal films deposited on their back sides. As the two films contact and separate, the alternative potential will drive electrons in the external load to flow back and forth. The polytetrafluoroethylene (PTFE) and polydimethylsiloxane (PDMS) are widely used polymer films of electronegativity in the TENG. Due to the nature of insulator-to-insulator or insulator-to-metal contact interface, the internal impedance of TENG is generally high. In order to enhance the output power, integration of piezoelectric PVDF thin film with other triboelectric functional layers to form a hybrid energy harvester in one device has been demonstrated^70^,^71^. However, both piezoelectric and triboelectric mechanisms have high internal impedance which limits the practical application of this kind of hybrid energy harvester to supply power to low load impedance scenarios.

Electromagnetic energy harvesters with low internal impedance collect the energy and generate current from coils due to the variation of magnetic flux induced from the movement of a permanent magnet. Mitcheson *et al*. have compared the performance limits of the three MEMS energy harvesting mechanisms^72^,^73^. It suggests that piezoelectric energy harvesters outperform electromagnetic energy harvesters at low frequency, and the electromagnetic mechanism is favourable in the high-frequency range. Piezoelectric energy harvesters usually generate high voltages and lower current, electromagnetic energy harvesters tend to produce relatively low AC voltage, and the voltage output is decreased when the size scales down. For applications with low vibration frequency, integration of piezoelectric and electromagnetic (or triboelectric and electromagnetic) to form a hybrid EH can have complimentary output, i.e., high voltage and high internal impedance from piezoelectric (or triboelectric) mechanism, and small voltage and low internal impedance output from electromagnetic mechanism. Thus it is a promising approach to increase output power based on hybrid energy harvesting mechanisms^74^,^75^,^76^,^77^,^78^,^79^,^80^,^81^.

Currently, most of the VEHs work on linear resonant system. But the overall energy output using such system is very limited and can only harvest peak power in narrow band of frequencies^81^. They are highly inefficient in terms of energy harvesting capability when frequency of vibration differs from one source to another, even slightly from the designed resonance frequency of a VEH. This is not desirable as ambient mechanical vibration exists randomly over wide range of frequencies from 30 Hz to 200 Hz. Thus, wide operation frequency range is a critical feature of VEHs. Several approaches have been reported to widen the operation frequency range^82^,^83^,^84^,^85^,^86^,^87^,^88^,^89^,^90^. One approach is to assemble an array of resonant structures together, which are excited at their own natural frequencies. Sari *et al*. have demonstrated a wideband energy harvester ranging from 4.2–5.0 kHz by implementing multiple cantilevers of different natural frequencies^82^. Yang *et al*. have reported a multi-frequency electromagnetic energy harvester consisting of three permanent magnets and corresponding to the resonant frequencies of 369 Hz, 938 Hz and 1184 Hz^83^. However, the bulkiness and complexity of the device overshadows its advantage in broadband behavior. Some other methods use multimodal energy harvesting^84^, spring non-linear stiffening^85^, frequency up-conversion^40^ and other techniques^86^ to widen the operation bandwidth. For example, the spring nonlinearity is introduced to broaden the bandwidth through several mechanisms such as magnetic levitation, non-linear stiffness and piezoelectric coupling^87^. Non-linear polymer based spring is another approach to widen the bandwidth but could only achieve around 20 Hz^88^.

Compared to other reported broadband techniques, frequency broadening by non-linear stiffening is relatively easier to realize. One main challenge is non-engagement of stoppers and therefore negligible output from triboelectric energy harvester due to non-contact of triboelectric layers at low accelerations^89^. It also leads to non-triggering of bandwidth broadening property. Another main challenge is to avoid mechanical energy loss incurred due to impact between moving mass and stoppers at high accelerations. Alternatively, in multimodal energy harvesting, the multiple modes of vibration can effectively widen the frequency bandwidth by merging multiple resonant peaks into one over a wide frequency range. These harvesters suffer mainly due to small displacement obtained at higher resonant modes compared with primary resonant mode^90^. Moreover, triboelectric output is very limited at low acceleration due to non-contact of charge generating layers of two triboelectric materials. Considering the above problems here, we propose a broadband and hybrid energy harvester (B-HEH) which overcomes the stated challenges with its distinctive design. Firstly, we utilized a soft polymer spring exhibiting closely located mode frequencies (1^st^ and 2^nd^ resonant mode) to widen the operating bandwidth at low acceleration. In this acceleration range (<0.5 g), the electromagnetic mechanism can harvest reasonable output even if oscillation amplitude is very small at higher modes. Thus, at a higher mode, i.e., the 2^nd^ resonant mode, combined output from triboelectric and electromagnetic mechanisms increases the overall B-HEH performance. Secondly, the mechanical energy loss during impact of a movable magnet, i.e., inertial mass, to top/bottom electrodes at high acceleration is reduced because part of this lost energy is scavenged and transformed into triboelectric output by integrating triboelectric electrodes as the top/bottom layer of the B-HEH. With adequate acceleration for the inertial mass to come in contact with top/bottom layer, the engagement with such stopper layer introduces non-linear stiffening. Due to contact-electrification on triboelectric layers, equal and opposite charge is generated and then collected by two electrodes utilizing freestanding mode of triboelectric mechanism^91^. The fabricated device can harvest over wide range of frequency from 50 to 130 Hz over widespread range of accelerations (0.1 g–2.0 g). The polymer spring fabricated using PDMS has been used to obtain nearby multimode frequencies. Compared with other reported HEHs^74^,^75^,^76^,^77^,^78^,^79^,^80^,^81^, the proposed B-HEH can operate under wide range of frequencies with its unique design, while it generates complementary output from electromagnetic and triboelectric mechanisms. Furthermore, this B-HEH design addresses two major objectives: firstly, the soft polymer spring with non-linear stiffness benefits in operation bandwidth widening, and secondly, hybrid mechanism increases the device performance over the attained frequency range.

## Results

### Device Configuration

**Figure 1:**
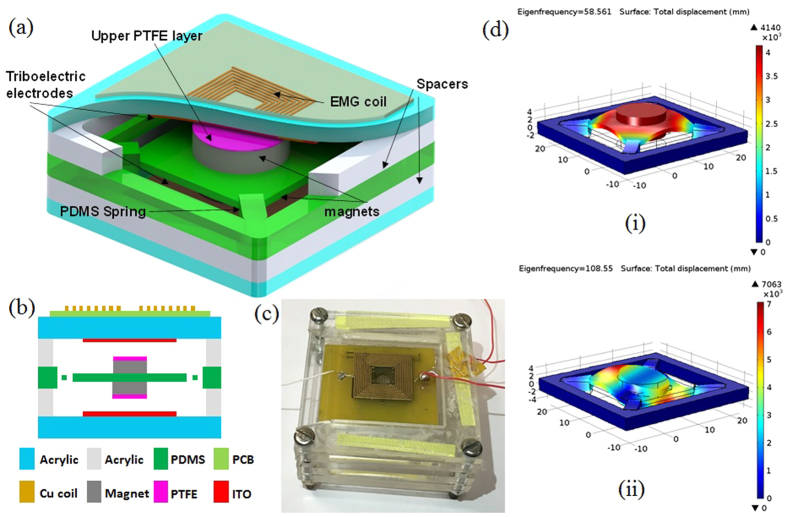
(**a**) Schematic drawing of broadband and hybrid energy harvester (B-HEH). (**b**) Cross sectional view of the device. (**c**) Prototype image of fabricated device. (**d**) FEM simulation using COMSOL.

Figure 1(a) and (b) show the device schematic drawing of B-HEH with integrated triboelectric energy harvester (TEH) and electromagnetic energy harvester (EMEH). The B-HEH consists of an electromagnetic coil, a pair of NdFeB magnets, a polymer spring structure, triboelectric materials and electrodes. The TEH consists of two PTFE layers and two Indium tin oxide (ITO) electrodes. The EMEH consists of fixed coil and moving magnet as proof mass for the resonant system suspended through PDMS spring structure as shown. A pair of NdFeB magnets are stacked on the PDMS stage which can oscillate along z-axis with any mechanical shock/vibration present in environment. The proposed design generates simultaneous electrical output from the electromagnetic coil and triboelectric energy harvester over broad range of frequencies. The mechanical spring structure used for the device is fabricated using PDMS polymer. The spring is fabricated using casting by a metal mold. The mathematic model of polymer spring system is shown in supplementary.

We design the spring dimensions and proof mass so as to achieve designed resonance frequency of 50–130 Hz for the PDMS spring. Figure 1(c) shows the fabricated prototype used for testing which consists of PDMS spring and stage structure, a PCB coil, a pair of NdFeB magnets, triboelectric materials, electrodes and acrylic case for housing. The acrylic substrates are cut by laser into 50 mm × 50 mm and placed as supporting case for soft PDMS springs. The moving PDMS stage with magnets serves as proof mass for spring-mass system. The EMEH consists of a fixed coil and two NdFeB magnets mounted on PDMS central platform as shown. The PDMS spring and rectangular stage structure hold the magnets which are stacked together. The detailed dimensions of the spring structure and different components are given Table 1. A modal analysis of the resonant structure was studied using finite element method (FEM) and simulated in COMSOL software. Figure 1(d) presents the first two resonant modes of the spring. Using simple elastic model, the primary resonance frequency is observed at 58 Hz for PDMS spring. The springs arms (7.07 × 3 × 3 mm^3^ each) and central platforms (20 mm × 20 mm) are fabricated with the flexible PDMS polymer. The top and bottom cylindrical NdFeB magnets (3.9 g × 2) of thickness 4.5 mm and diameter 12 mm are stacked together on the polymer stage as shown. The surface flux density of NdFeB magnets is 0.3 T and as the distance increased from the surface magnetic flux density falls rapidly. Since the magnetic flux density is high near the surface, the gap between coil and magnet should be as minimum as possible as given by expression^81^ as given by following equation:


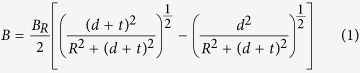


where *B*_*R*_ is the residual magnetic flux density, *d* is the distance of the point from magnet, *R* and *t* are the radius and thickness of the magnet. The coil for the EMEH consists of multilayer copper on FR4 PCB substrate. The PCB thickness is 2 mm containing 12 layer of copper coils with ten turns for each coil. The coil area is designed for 15 mm × 15 mm with resistance of 15 Ω which consist of copper of thickness, width and spacing of 35 μm, 254 μm and 254 μm, repectively.

For TEH, the freestanding mode of triboelectric mechanism is realized by having PTFE dielectric layers of dimension 20 mm × 20 mm × 250 μm attached on magnets moving between two electrodes. The dielectric PTFE layer is negatively charged upon contact with metal electrode. The metal layers serve as positive triboelectric charged layers and electrodes for collecting the generated charge. The ITO coated PET sheet of dimension 20 mm × 20 mm × 200 μm are attched on top and bottom acrylic substrates as electrodes. The size of whole device is 5 cm × 5 cm × 2.5 cm.

### Working Principle

The volatge generation of B-HEH is tested with two modes of mechanical input as sinusoidal vibration and tapping. The EMEH works on Faraday’s law of electromagnetic induction, which develops the electromagnetic force with change of rate of flux passing through the coil. For TEH, the two triboelectrically opposite materials (PTFE and ITO) develop equal and opposite charge when come in contact. Further, the working modes of B-HEH can be divided in to two modes as shock/tapping and resonant mode. The electromagnetic output is extracted directly from the coil while the TEH ouptut is collected using freestanding electrodes.

**Figure 2:**
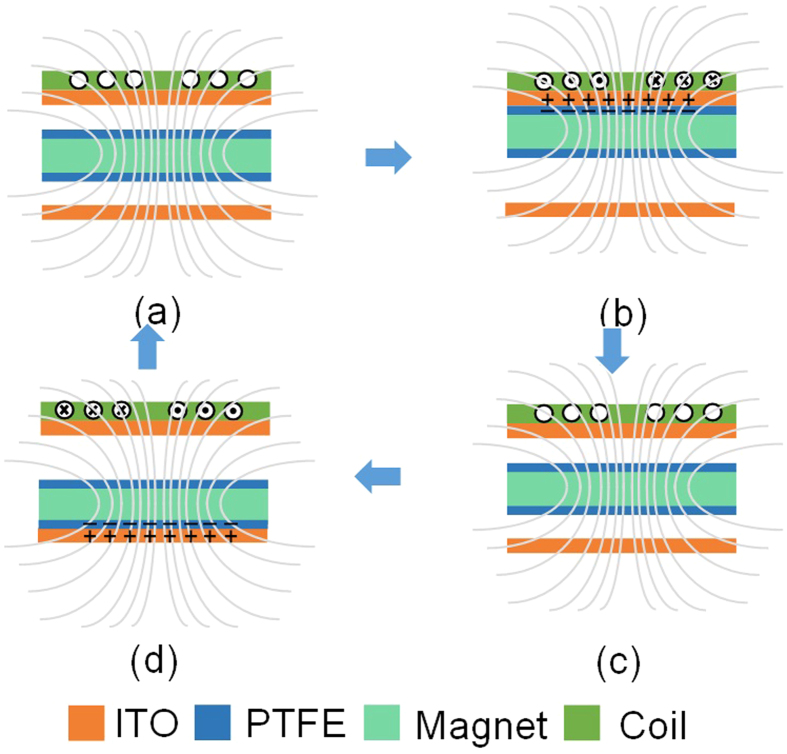
Steps for voltage generation using triboelectric and electromagnetic mechanism in four steps.

The combined energy harvesting principle of the device is presented in schematic shown in Fig. 2. The triboelectric charge generation can be divided into four sub-states as shown in Fig. 2(a–d). Initially the proof mass remains in the middle position at an equal distance from the upper and lower triboelectric layer. As, mechanical vibration is given to the device in z-axis, the proof mass moves to contact with the upper layer or tend to contact depending upon applied acceleration. In Fig. 2(a), the device is in original state where the proof mass is suspended without any contact with either of the surface. When it is is mechanically excited in z-axis, the PTFE coated suspended proof mass comes in contact with top ITO, generating the triboelectric charge as shown in Fig. 2(b). Simultaneously, magnetic flux through the EMEH coil increases leading to negative current flow in the coil. In next step, the dielectric crosses middle position and with negative accleration comes in contact with bottom ITO as shown in Fig. 2(d). Simultaneously, the magnetic flux linkage in EMEH coil changes from maximum to minimum leading to positve current flow as per Lenz’s law. Thus, in one compelete oscillation, the device generates triboelectric output and simultaneously generates electromagentic peak volatge during the sub-states in Fig. 2(b,d) when the flux linkage throught the coil is maximum and minimum. During resonant mode, at lower acceleration (<1 g), the triboelctric layers may not have proper contact with the opposite triboelctric layers leading to very small surface charge densities, but the device still generates reasonable electromagentic output.

### Energy harvesting in resonant mode

**Figure 3:**
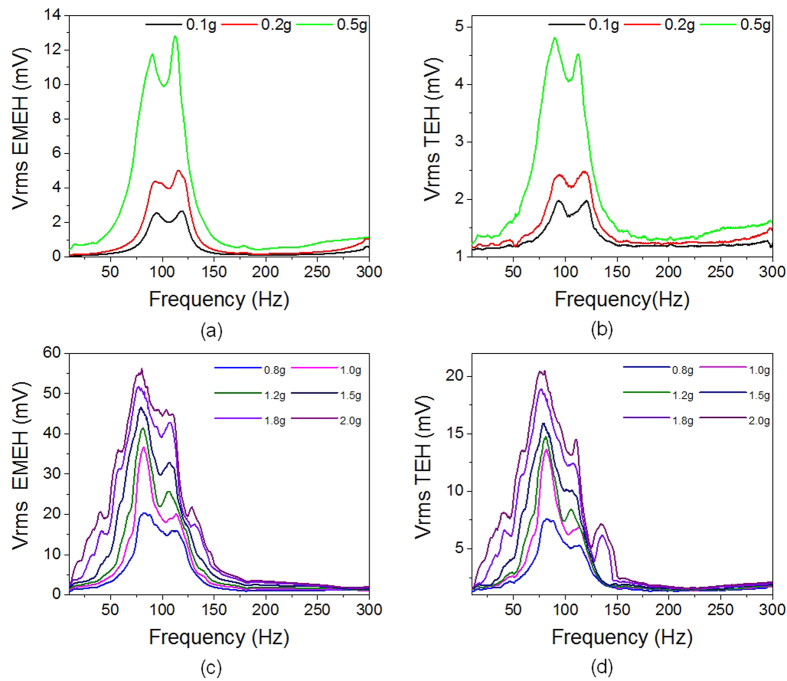
(**a,b**) Frequency spectrums for electromagnetic and triboelectric output voltage at 0.1 g, 0.2 g and 0.5 g exhibiting multimodal behavior. (**c,d**) Frequency spectrum for electromagnetic and triboelectric output voltage at accelerations higher than 0.5 g; non-linear stiffening effect can be observed due to engagement of mechanical stoppers for different accelerations from 0.5 g to 2 g.

Using setup as given in Supplementary information (Figure S1), the B-HEH is mounted on the shaker’s stage and sinusoidal frequency sweep is applied from 10 to 300 Hz at different accelerations ranging from 0.1 g to 2.0 g. Figure 3 shows the frequency spectrum output of both TEH and EMEH under different accelerations. It is apparent from the frequency spectrum that the device exhibits a broadband characteristic over wide range of frequency. It can also be seen that the broadband characteristic of the energy harvester spreads from 50 Hz to 130 Hz centered around 82 Hz. The peak RMS voltages for TEH and EMEH are found to be 20 mV and 55 mV, respectively, measured by using DSA (digital signal analyzer) at excited acceleration of 2 g. After achieving peak voltage at 82 Hz, the voltage decreases gradually with frequency and then a second resonant peak is observed at 110 Hz. In Fig. 3(a,b), the output from TEH is less at low acceleration (<0.5 g) as the triboelectric layers are unable to contact each other. When the B-HEH was excited with higher acceleration (>0.5 g), the attached dielectric layers on proof mass are able to contact with ITO electrodes, generating high triboelectric charges. To judge the criteria for broadband behavior, full width half maximum (FWHM) is adopted as the parameter. It is worth to note that the operation bandwidth of 40 Hz is achieved using soft PDMS spring even at such a low excitation of 0.1 g. It is the combination of non-linearity from spring stiffness and nearby mode frequencies of PDMS spring which enables the device to harvest energy over wide range of frequency (68 Hz at 2.0 g). For PDMS spring, the primary and secondary resonant frequencies are observed at 82 Hz and 110 Hz. At low accelerations (0.1 g to 0.5 g), the two nearby modes can be clearly observed. At these accelerations, each resonant peak has narrow frequency operating area similar to linear resonant system as shown in Fig. 3(a,b). When the device is subjected to higher acceleration, frequency broadening effect can be observed in Fig. 3(c,d). This is due to non-linear stiffness of spring introduced by engagement of mechanical stoppers, i.e. the acrylic substrates on top and bottom sides. The non-linear stiffening effect is not observed at low acceleration due to insufficient displacement experienced by proof mass. But, the broadband behavior is still achieved at low acceleration by merging two adjacent resonant modes into one due to closely located mode frequencies of polymer spring.

On the other hand, significant increment in triboelectric output is observed in data measured at acceleration above 0.5 g. The impact of inertial mass to acrylic substrates with ITO electrodes leads change in resonance behavior of system above 0.5 g, and to generate triboelectric output because of contact-electrification. By leveraging this TEH, the loss due to mechanical impact is partially transformed into energy via triboelectric output.

The time dependent voltage and current waveforms from TEH are given in Supplementary information (Figure S2). The voltage output from TEH is sinusoidal due to applied sinusoidal excitation. The short circuit current has asymmetric alternating characteristic; the positive and negative peak depicts the contact and release of suspended dielectric with ITO. The TEH output is relatively small due to impedance mismatch between the TEH and DSA. The open circuit voltage from TEH is measured with 100 MΩ probe, the peak-peak open circuit voltage and short circuit current is found to be 9.5 V and 70 nA, respectively, at 2 g at dwell frequency of 82 Hz. The output from TEH is small at low acceleration (<0.5 g) due to non-contact between two triboelectric sides. When the B-HEH is excited with higher acceleration, the spring mass system enables the attached dielectric to contact ITO electrodes on the acrylic substrates. The peak power output from TEH is found to be 0.166 μW.

The EMEH output voltage is 55 mV RMS at acceleration of 2 g and output RMS power is 50 μW at optimum load of 15 Ω. Considering the half peak voltage, at 2 g EMEH can provide RMS voltage larger than 28 mV from 50 Hz to 118 Hz displaying wide operation frequency range of 68 Hz. Similarly, the EMEH can generate peak RMS voltage 6 mV, 36 mV and 46 mV with bandwidths of 30 Hz, 40 Hz and 55 Hz at 0.1 g, 0.8 g and 1.5 g, respectively. The RMS power for EMEH at these accelerations is at 0.6 μW, 21.6 μW and 35.27 μW at optimum load of 15 Ω.

**Figure 4:**
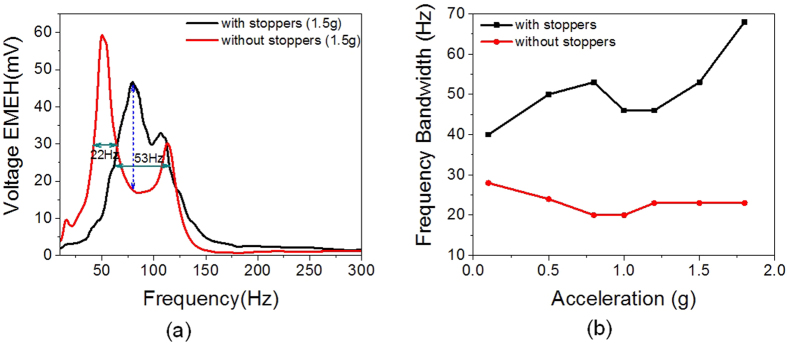
Broadband behavior of B-HEH using FWHM parameter. Comparison of frequency response with and without stoppers. (**a**) Frequency response of devices with and without stoppers at 1.5 g. (**b**) Comparison in attained bandwidth at different accelerations.

Thus, the polymer spring fabricated using PDMS introduces nearby mode frequencies while the stopper brings non-linear stiffness into frequency spectrum. Here, the stopper serves dual purpose of utilizing the mechanical impact to generate triboelectric charge structure along with non-linear stiffening of PDMS spring contributing to increased output and high operating bandwidth. To have proper comparison in terms bandwidth improvement, the harvester is tested without any stoppers and frequency spectrum for electromagnetic output is recorded at different accelerations. Figure 4(a) shows the frequency spectrum of EMEH output voltage with and without stoppers measured at 1.5 g. The comparison plot for attained bandwidth at different accelerations is shown in Fig. 4(b). From the figure it can be observed that the bandwidth increased from 23 Hz to 68 Hz by using stoppers at 1.8 g. Further, the percentage increase in bandwidth is calculated by subtracting the upper and lower frequency using FWHM. As observed from the graph the percentage increase in bandwidth is found to be 295% at 1.8 g by introducing mechanical stoppers.

### Energy harvesting in non-resonant mode

**Figure 5:**
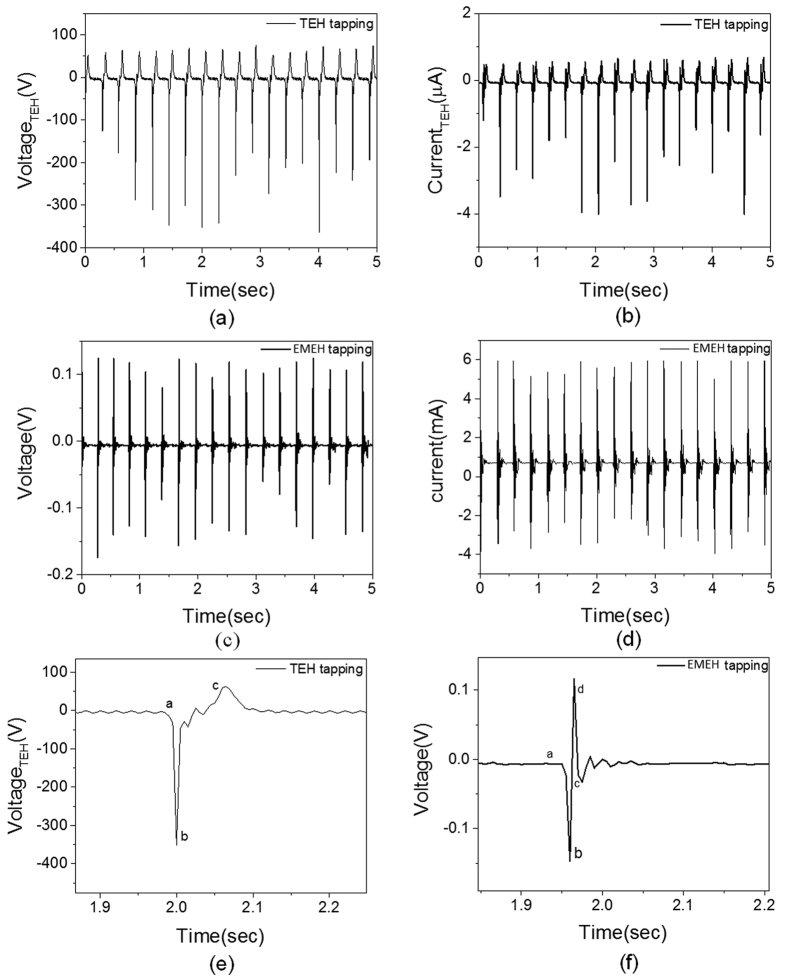
Non-resonant mode characterization. (**a**) Volatge output from TEH. (**b**) Short circuit current from TEH. (**c**) Volatge output from EMEH. (**d**) Short circuit current from EMEH. (**e,f**) Step by step explantion of volatge generated from TEH and EMEH, repectively.

**Figure 6:**
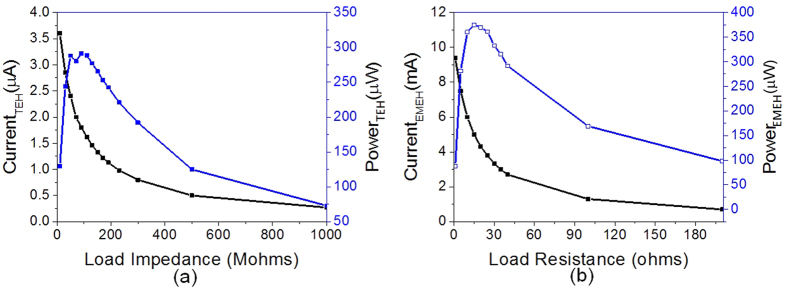
Power spectrum for Non-resonant mode. (**a**) TEH output short circuit current and power with resistances (0–200 MΩ). (**b**) EMEH output short circuit current and power with resistances (0–200 Ω).

The B-HEH is also tested with hand tapping and corresponding output from EMEH and TEH is shown in Fig. 5. Under hand tapping, the TEH can generate peak open circuit voltage and short circuit current of 200 V and 0.5 μA, respectively. Figure 5(a,b) shows the output voltages and currents due to hand-tapping from TEH. When the device is tapped, the PTFE on inertial mass makes contact with ITO electrode resulting in triboelectric output. The electrical output from EMEH and TEH are divided into 4 modes (i–iv), as described in Fig. 5(e,f). The contact-electrification at higher force led to more charge generation, resulting in higher electrical output compared to resonant mode. The output voltage and current from EMEH are shown in Fig 5(c,d). The electromagnetic output achieves peak voltage at the tapping instant due to sharp change in magnetic flux. Thereafter, the system undergoes damped vibration leading decreased output from EMEH. The EMEH generates a large current due to its low internal impedance. The EMEH and TEH provide complementary output which broadens its application for both high and low impedance electrical loads. For investigating the power spectrum, the external load is connected with the EMEH and TEH output electrodes. For TEH, short circuit is measured with external load ranging from 0 to 200 MΩ and calculated power is plotted in Fig. 6(a). The peak power generated by TEH in tapping mode is 300 μW at optimum impedance of 75 MΩ. Similarly, the short circuit current from EMEH is measured at external load from 0 to 200 Ω. The short circuit current and the power spectrum of EMEH with hand-tapping is plotted in Fig. 6(b). The EMEH can generate maximum power of 375 μW at optimum load of 15 Ω. The maximum power density during tapping mode is found to be 4.8 μW/cm^3^ for TEH and 6 μW/cm^3^ for EMEH.

### Characterization as a triboelectric accelerometer

The voltage output of a triboelectric device is given as:


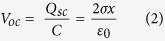


the displacement of proof mass is found to be linear with triboelectric output voltage, and using the expression given by Hook’s law. *F* = *kx* = *ma*, the z-axis when plotted with acceleration, a linear relationship is observed that acceleration is linearly related to triboelectric output voltage and thus acceleration sensitivity is given by:


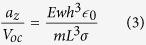


**Figure 7:**
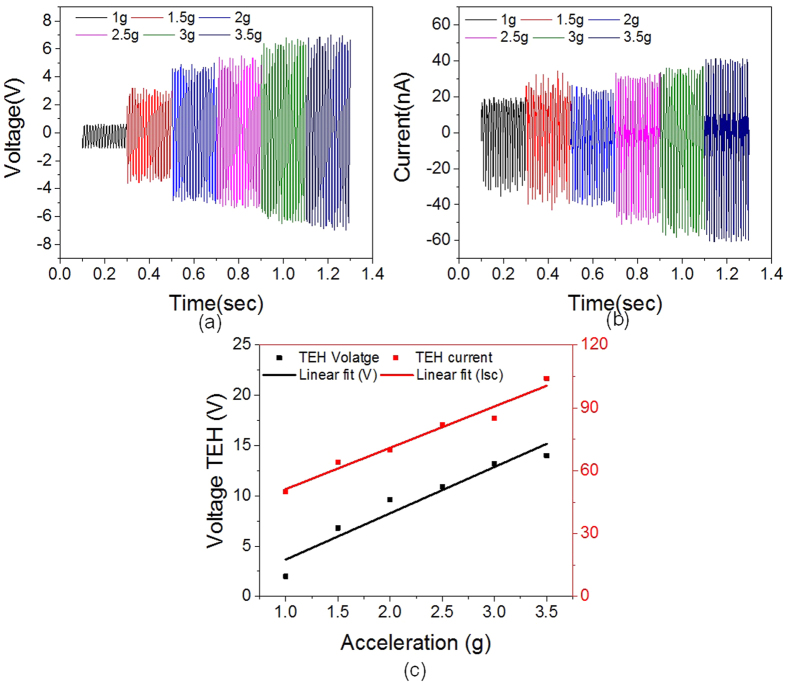
Characterization as a triboelectric accelerometer. (**a**) TEH output voltage waveform at different sinusoidal acceleration measured at dwell frequency of 82 Hz. (**b**) TEH short circuit current waveform at different sinusoidal acceleration measured at dwell frequency of 82 Hz. (**c**) Linear relation observed between TEH outputs and applied acceleration with voltage and current sensitivity of 4.7 Vg^−1^ and 19.7 nAg^−1^, respectively.

To demonstrate the practical uses of device, we utilize the triboelectric output as self-generated acceleration signals. Using electromagnetic shaker, sinusoidal acceleration is applied on the B-HEH and time-dependent output voltage and short circuit current is measured. The output voltage for B-HEH at different accelerations shown in Fig. 7(a) is measured with impedance of 100 MΩ. The short circuit current is measured using a low noise SR570 current pre-amplifier. With high acceleration from 1 g to 3.5 g, the triboelectric output follows a linear relationship with acceleration as shown in Fig 7(c). We can conclude from that the triboelectric output voltage and short circuit current from resonant mode has a linear relationship with acceleration. Equation (2) predicts such linearity between voltage output and acceleration. The device can be used to detect acceleration ranging from ±1 g to ±3.5 g (g = 9.8 m/s^2^) with voltage sensitivity of 4.7 Vg^−1^ and current sensitivity of 19.7 nAg^−1^. The fitting curve of output voltage and output current is linearly fitted with the correlation coefficient of 0.96 and 0.98. The triboelectric output measured at acceleration below 1 g is very less compared to results above 1 g, this is because non-contact of two triboelectric layers leading to almost zero generated charge density. From the Fig. 1(d–i), it shows that the inertial mass moves up and down in the 1^st^ resonant mode. Thus the above data suggests that this device could be used as a z-directional accelerometer for applications with acceleration above 1 g by measuring the triboelectric voltage or triboelectric current output. More comprehensive characterization would be required in future in order to make fair comparison to current commercial accelerometers.

## Discussion

In summary, a broadband and hybrid energy harvester using the triboelectric and electromagnetic mechanisms, i.e., B-HEH, is fabricated and tested. Firstly, the multimodal energy harvesting is achieved using soft PDMS polymer springs with non-linear stiffness benefiting in widen operation frequency bandwidth. Secondly, integration of electromagnetic and triboelectric mechanisms increases the overall device performance over the obtained bandwidth. To enhance the output, TEH and EMEH are integrated in the B-HEH to achieve complementary output functionality during tapping and resonant mode, where power output of 50 μW and 166 nW is obtained in resonant mode, respectively. During tapping mode testing, B-HEH can harvest 300 μW from TEH and 375 μW from EMEH, respectively. Compared to linear resonant system, the proposed device has advantage of wide operating range to harvest ambient mechanical vibration. The proposed PDMS polymer spring introduces the multimode resonant behavior at low accelerations (<0.5 g) contribute to widening of bandwidth. Also, the fixed triboelectric layers act as mechanical stopper for spring mass system brings non-linear stiffening into the device at higher acceleration. These two effects help to achieve wide bandwidth up to 68 Hz. When non-linear stiffening is used, the obtained bandwidth increases from 23 Hz to 68 Hz with percentage increment of 295% at 1.8 g. It enables the B-HEH to harvest mechanical vibration available ranging from 50 Hz to 130 Hz with center frequency at 82 Hz. The device performance is compared in Table 2 with other reported energy harvesters based on obtained bandwidth and energy harvesting performance. Our device has wide frequency range of 68 Hz at 2 g. Even at low acceleration of 0.1 g, bandwidth is found to be 40 Hz which is much higher compared to previous reported devices. Further, the triboelectric output is demonstrated for acceleration sensor with voltage and current sensitivity of 4.7 Vg^−1^ and 19.7 nAg^−1^. The output performance of the device can be further improved by optimizing the dimensions of PDMS spring and gap between the dielectric and metal electrode in the future. The proposed device can be potentially used for self-powered shock detection in automobiles and other shock sensitive applications.

## Methods

### Fabrication fo PDMS Spring

The four arm PDMS spring is fabricated through casting process using metal mold. The metal mold (50 mm × 50 mm × 5 mm) iss fabricated using Steel (SUS304). Initailly, metal mold is cleaned with IPA and non-adhesive coating is applied on it to reduce the adhesion between polydimethylsiloxane (PDMS) and metal mold during peeling off process. Thereafter, the PDMS mixture (10:1) is pored on to the metal mold and degassed in dessicator for 2 hrs to let the PDMS fill each and every corner of metal mold strucutre. The prepared sample is then cured at 100 °C for 1 hr and the PDMS spring strucutre is peeled off from the metal mold. The fabricated PDMS spring is then assembled and used for the spring mass resonant system.

### Device assmbling

Figure 1(b) shows the fabricated prototype used for testing that consists of PDMS spring and stage structure, a PCB coil, a pair of NdFeB magnets, triboelectric materials, electrodes and acrylic case for housing. The acrylic substrates are cut by laser into 50 mm × 50 mm and placed as supporting case for soft PDMS springs. The fabticated PDMS srping with stacked magnets are assembled with use of acrylic spacers (thickness of 5 mm) to create gap of 2 mm between proof mass and stoppers.

### Frequency Response Measurement

The output performance of B-HEH is measured under two modes of mechanical energy. At first, the sinusoidal vibration is applied on the device at different accleration using an electromagnetic shaker and the triboelectric and electromagnetic volatges are measured repectively using signal analyzer. Also, the device is subjected to shock such as hand tapping, and outputs from THE and EMEH are measured. The testing setup employed for obtaining the frequency spectrum of device is given in Supplementary information (Figure S1). The setup mainly consists of a vibration shaker, dynamic signal analyser (DSA), a power amplifier, a vibraton controller, and an accelerometer. Device is tested using shaker (Brüel & Kjær vibration exciter type 4809) and vibration controller (Type 7542). The signal from the DSA (Model no: HP 35670A) is applied to shaker (Brüel & Kjær vibration exciter type 4809) and output from energy harvester iss measured using system analyzer. To form the control loop for vibration controller, an accelerometer is employed to get the actual applied accleration on the device and feedbacked to system. For obtaining the frequecy response of device, we perform frequency sine sweep on the B-HEH. The signal from the EMEH and TEH is measured using different ports. An alumunium stage is used to fix the hybrid energy harvester in shaker and time dependant output iss taken using Digital oscilloscope.

### Time dependant Voltage and Current measurement

For voltage measurement, B-HEH is connected with a 100 MΩ probe and measured by DSOX3034A oscilloscope. The short circuit current was measured by using a low noise SR570 current pre-amplifier.

## Additional Information

**How to cite this article**: Gupta, R. K. *et al*. Broadband Energy Harvester Using Non-linear Polymer Spring and Electromagnetic/Triboelectric Hybrid Mechanism. *Sci. Rep.* **7**, 41396; doi: 10.1038/srep41396 (2017).

**Publisher's note:** Springer Nature remains neutral with regard to jurisdictional claims in published maps and institutional affiliations.

## Supplementary Material

Supplementary Information

## Figures and Tables

**Table 1:** Structural parameters and material properties.

Parameter	Description	Value
*E*	PDMS Young’s Modulus	550 kPa
*l*	Length of PDMS spring leg	7.07 mm
*w*	Width of PDMS spring leg	3 mm
*h*	Height of PDMS spring leg	3 mm
*A*_*s*_	PDMS stage area	2 cm × 2 cm
*R*	Radius of NdFeB magnet	6 mm
*t*	Thickness of NdFeB magnet	4.5 mm
*N*	Turns of EMG coil	120
*L × B × H*	Dimension of whole device	4 cm × 4 cm × 2.5 cm
*d*_1,_ *d*_2_	Gap between two triboelectric layer	1 cm

**Table 2:** Comparison of present work to other published work with Operating Bandwidth.

Approaches used for increasing Bandwidth	Reference	Mechanisms	Acceleration (g)	Center frequency (Hz)/Excited frequencies (Hz)	BW range (Hz)	Power	Power density
Multimodal Energy Harvesting + Hybrid mechanism	Challa *et al*.^90^	Electromagnetic + Piezoelectric	—	21.6 Hz	—	332 μW	9.5 μW/cm^3^
	Tadesse *et al*.^84^	Electromagnetic + Piezoelectric	35 g	20 Hz, 100 Hz	—	250.25 mW	2.66 mW/cm^3^
Non-linear Stiffening	Liu *et al*.^39^	Piezoelectric	0.6 g	39 Hz	18 Hz	0.88 μW	159.4 μW/cm^3^
	Jeon *et al*.^86^	Triboelectric	17.5 g	11 Hz	22 Hz	6.52 μW	0.023 μW/cm^3^
	Dhakar *et al*.^57^	Triboelectric	1.6 g	30 Hz	22.05 Hz	0.91 μW	0.23 μW/cm^2^
Multimodal + Hybrid + Non-linear Stiffening	This Work	Triboelectric + Electromagnetic	2.0 g	80 Hz	68 Hz	50.2 μW	0.8 μW/cm^3^
