# Supplementary material for: Broadband Energy Harvester Using Non-linear Polymer Spring and Electromagnetic/Triboelectric Hybrid Mechanism
Source: Sci Rep. 2017 Jan 25;7:41396. doi: 10.1038/srep41396 (PMC5264648; doi:10.1038/srep41396)
Supplement: Supplementary Information [file srep41396-s1.doc]

**Supplementary Information**

**Broadband Energy Harvester Using Non-linear Polymer Spring and Electromagnetic/Triboelectric Hybrid Mechanism**

**Rahul Kumar Gupta^1,2^, Qiongfeng Shi^1,2^, Lokesh Dhakar^1,2^, Tao Wang^1,2^, Chun Huat Heng^1,2^ and Chengkuo Lee^1,2,3*^**

**^1^Department of Electrical and Computer Engineering, National University of Singapore, 4 Engineering Drive 3, Singapore 117576**

**^2^Center for Intelligent Sensors and MEMS (CISM), National University of Singapore, Singapore**

**^3^NUS Graduate School for Integrative Science and Engineering, National University of Singapore, Singapore 117456**

***Corresponding author: Chengkuo Lee**

**E-mail:** [**elelc@nus.edu.sg**](mailto:elelc@nus.edu.sg)

**TEL: (65)6516-5865; FAX: (65)6779-1103**

**Mathematic model of polymer spring system.**

The resonance frequency for the spring can be expressed as:

|  | 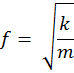 | (S1) |
| --- | --- | --- |

where *k, m, f* are the linearized spring constant of PDMS structure, proof mass and resonance frequency of system respectively. Using beam theory, the spring constant *k* can be given as:

|  | 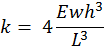 | (S2) |
| --- | --- | --- |

where *E* is the Young's modulus of the beam material, *w*, *h* and *L* are the width, thickness and length of the beam, respectively. Thus the resonance frequency *f* can be re-written as:

|  | 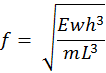 | (S3) |
| --- | --- | --- |

The equation of motion for the proof mass can be expressed by second order of differential equation is given by:

|  | 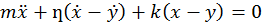 | (S4) |
| --- | --- | --- |

where η is the damping coefficient, *ω* is the excitation frequency,
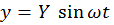
is the base excitation and x is the displacement of suspended mass.


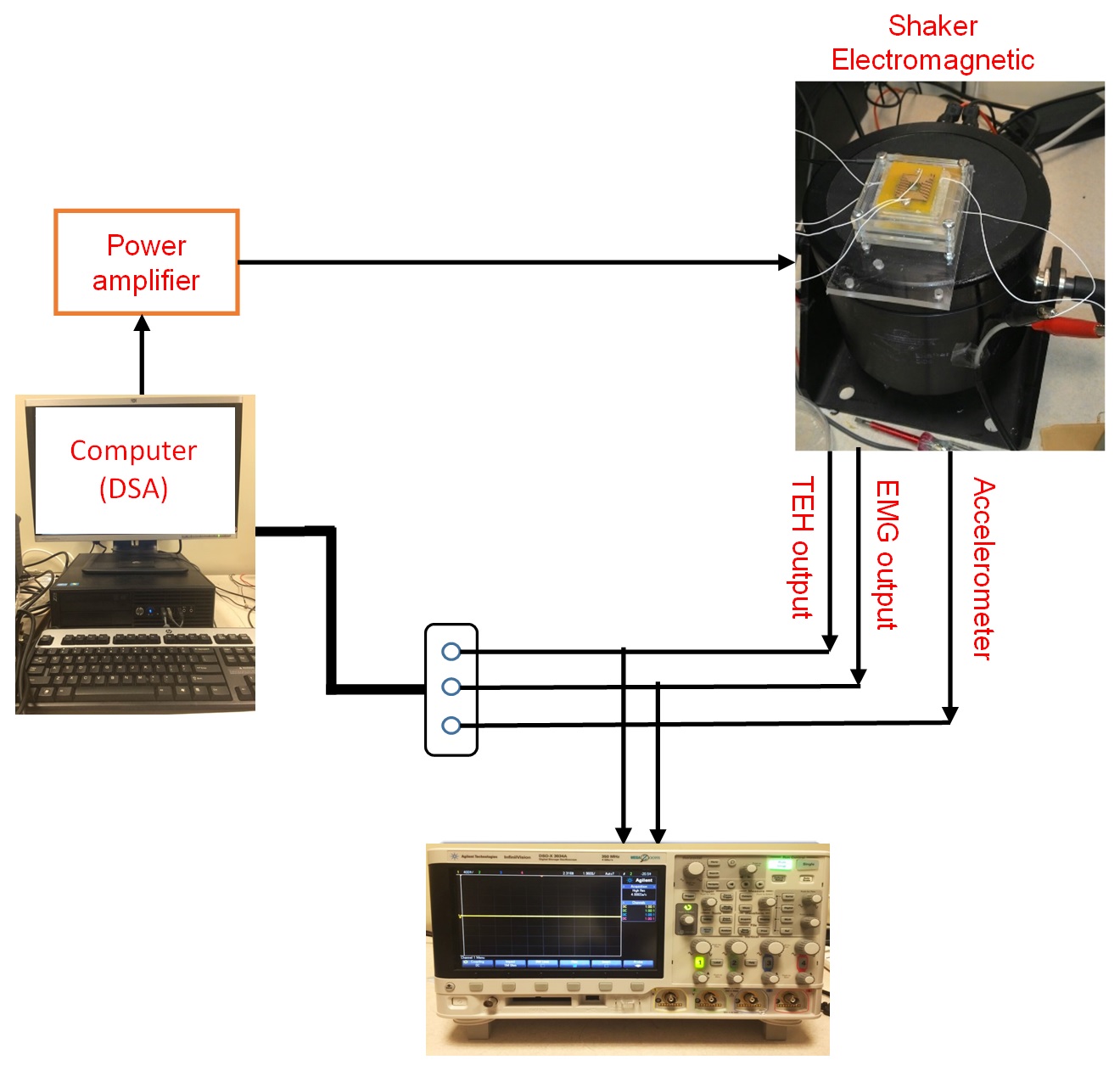


Figure S1. Experimental setup for obtaining frequency characteristics of the proposed B-HEH.

Figure S2. Triboelectric output measured with high impedance probe. (a) TEH output voltage waveform measured at acceleration 2g and dwell frequency of 82Hz. (b) TEH short-circuit current waveform measured at acceleration 2g and dwell frequency of 82Hz.
